# Network hubs affect evolvability

**DOI:** 10.1371/journal.pbio.3000111

**Published:** 2019-01-30

**Authors:** Jana Helsen, Jens Frickel, Rob Jelier, Kevin J. Verstrepen

**Affiliations:** 1 CMPG Laboratory of Genetics and Genomics, Departement Microbiële en Moleculaire Systemen (M2S), KU Leuven, Leuven, Belgium; 2 VIB Laboratory for Systems Biology, VIB-KU Leuven Center for Microbiology, Leuven, Belgium; 3 CMPG Laboratory of Predictive Genetics and Multicellular Systems, Departement Microbiële en Moleculaire Systemen (M2S), KU Leuven, Leuven, Belgium

## Abstract

The regulatory processes in cells are typically organized into complex genetic networks. However, it is still unclear how this network structure modulates the evolution of cellular regulation. One would expect that mutations in central and highly connected modules of a network (so-called hubs) would often result in a breakdown and therefore be an evolutionary dead end. However, a new study by Koubkova-Yu and colleagues finds that in some circumstances, altering a hub can offer a quick evolutionary advantage. Specifically, changes in a hub can induce significant phenotypic changes that allow organisms to move away from a local fitness peak, whereas the fitness defects caused by the perturbed hub can be mitigated by mutations in its interaction partners. Together, the results demonstrate how network architecture shapes and facilitates evolutionary adaptation.

## Abstract 2

Genes are organized into complex interaction networks, but it is unclear how network architecture affects evolution. This Primer explores a new study which uses experimental evolution to show how alterations in a gene central to a network affect evolutionary processes.

Every cellular process in an organism, from mitosis to metabolism, results from the integrated and coordinated activity of a specific subpart of its gene network. In its most simple form, a network can be visualized by a set of genes or gene products, linked together by pairwise interactions such as protein–protein or protein–DNA interactions. Biological networks typically show several levels of organization. Genes involved in related processes often share interaction partners and form smaller subnetworks or modules. The availability of today’s genome- and proteome-scale assays have made it possible to systematically map individual genes within the network, which allowed us to obtain an overview not only of how cellular regulation is organized but also of the precise function of individual genes and gene modules within the cell [1].

One striking conclusion from mapping cellular networks is that many genes only interact with a limited number of other genes, whereas a smaller subset of genes interacts with many other genes and therefore has a more central role in the gene network (so-called network hubs). Hubs are often predicted to be essential for an organism’s fitness, because any perturbation can affect many other genes. In line with this hypothesis, it has been shown that hubs are three times more likely to be essential than genes with fewer interaction partners [2]. Furthermore, there is a negative correlation between the connectivity of a protein and the growth rate of its knock-out strain [3]. Perturbations of network hubs are therefore expected to have major fitness consequences. However, it is less clear how the existence of hubs affects the evolvability of an organism (i.e., the ability of the organism to produce heritable—potentially adaptive—phenotypic variation) and whether hub genes evolve differently compared to peripheral genes. Does their essentiality and central role in the gene network lead to slower evolutionary rates, as often suggested? Indeed, previous studies found that the position of a gene within the network often correlates with its rate of evolution. For example, the number of interaction partners of a gene shows a negative correlation with its rate of evolution [4,5,6]. Because of this, evolution is thought to primarily occur through mutations in genes at the periphery of the gene network [7]. However, the strength of the correlation between the connectivity of a protein and its evolutionary rate differs between different studies. Some studies that rely on different interaction databases or that employ different analysis methods do not find significant correlations [3]. In part, such differences could be caused by confounding factors such as expression-level differences or be due to biased or low-quality data. Furthermore, there are many different types of networks, including genetic interaction networks, physical and protein–protein interaction networks, transcriptional regulatory networks, and metabolic networks. Therefore, results might also vary depending on the type of molecular network that is considered. For example, in a bacterial metabolic network, network topology showed little correlation with enzyme evolution [8]. In yeast, however, central transcription factors have been reported to evolve faster [9]. Because there is no general consensus regarding the role of hub genes in evolution, more experiments are needed to determine the role of network architecture in evolutionary biology.

In a new study, Koubkova-Yu and colleagues investigate how hubs affect the evolvability of yeast by studying how cells respond to perturbations in the network hub *HSP90* [10]. Hsp90 is a molecular chaperone and an exceptional hub gene because it is extremely abundant in cells [11] and it interacts with more than 10% of the yeast proteome [12]. It has been termed a “hub of hubs” because many of its clients are also network hubs within the interaction network [13]. Despite its central position in the interaction network, the authors present here that *HSP90* does show sequence divergence when comparing four yeast species separated by 50 to 270 million years of evolution. This raises the question of whether the hub gene itself evolved different functions in the different species. To test for this, the authors replaced *HSP90* in *Saccharomyces cerevisiae* with its orthologs from the three other yeast species and measured the growth of the resulting mutant strains. Although replacement with the two closest related *HSP90*s did not have strong fitness effects, replacement with the *HSP90* ortholog from *Yarrowia lipolytica* did result in severe growth defects.

Interspecies gene replacements can affect fitness in many different ways. The function of the genes might have diverged, the expression level can be suboptimal, the activity of the protein might be reduced, or the interactions with other genes could be changed [14,15]. Although the authors find no differences in expression levels and activity, the precise reason for reduced fitness was not further disentangled. However, *S*. *cerevisiae* cells with the *Y*. *lipolytica* ortholog showed improved growth in high-salt environments. *Y*. *lipolytica* is often isolated from hypersaline environments and has a higher salt tolerance than *S*. *cerevisiae*. Therefore, solely replacing *HSP90* resulted in a partial transfer of the salt-tolerant phenotype. A similar phenomenon was observed in *Escherichia coli*, in which expression of a chaperonin from the psychrophilic bacterium *Oleispira antarctica* resulted in improved growth at low temperatures [16]. Koubkova-Yu and colleagues propose that the altered fitness patterns might be explained by breakage of important interactions in the *HSP90* network [10]. Alternatively, it is also possible that Hsp90 from *Y*. *lipolytica* has an improved stability or functionality in hypersaline conditions. Because Hsp90 is a hub protein, improving its stability or functionality in a stressful environment could substantially improve growth because it may rescue the function of several important interaction partners. This would imply that, rather than being unchangeable obstacles, some hubs could be prime drivers of evolution.

Even if mutations in a hub could be adaptive in some specific environments, it is to be expected that the overall fitness effect is negative. Therefore, the authors asked whether cells with altered network hubs can adapt to overcome their fitness defects. More specifically, will cells generally evolve to restore the original Hsp90 function, or will they adapt by rewiring their gene network? To answer these questions, the authors repeatedly evolved originally isogenic populations of *S*. *cerevisiae* containing the *Y*. *lipolytica HSP90* ortholog for more than 2,000 generations. Strikingly, all lines quickly showed fitness increases, suggesting that a perturbed hub is not necessarily an evolutionary dead end. Furthermore, none of the evolved populations showed mutations in *HSP90* itself. Instead, adaptation was driven by mutations in one or more genes that are connected to each other by *HSP90*. As such, they showed that adaptation to an altered hub occurred by optimizing the subnetworks the hub is connected to and not by restoring the hub itself. These subnetworks were different between the populations, and as a result, the evolved lineages showed a large variety in their phenotypic profiles.

The observations of the authors are especially interesting when put into the context of fitness landscapes [17,18]. Previous simulations show that cells can reach different, and in some cases even higher, fitness peaks by starting off from a valley instead of from a local maximum [19]. The structure of the remaining gene network then determines which new fitness peaks are available for the evolving individual. Because hubs are involved in multiple processes and have a large number of interaction partners, the number of possibilities an organism has to reach another local maximum is higher when a hub is perturbed than when a gene at the edge of the network would be perturbed (Fig 1). In the fitness landscape, such dramatic changes translate into a large translocation, away from a (local) fitness peak, which in turn allows organisms to explore other parts of the landscape and find alternative fitness optima, which may in fact be higher than the original starting point. In addition, it is interesting to note that when they are far removed from fitness peaks, populations primarily evolve by fixing rare mutations that have a relatively large (positive) fitness effect [20], which can result in relatively large phenotypic variability between independently evolving populations [21]. This is indeed what the authors find in this study: many of the mutations resulted in strong fitness increases, but they are different in the different evolved populations.

**Fig 1:**
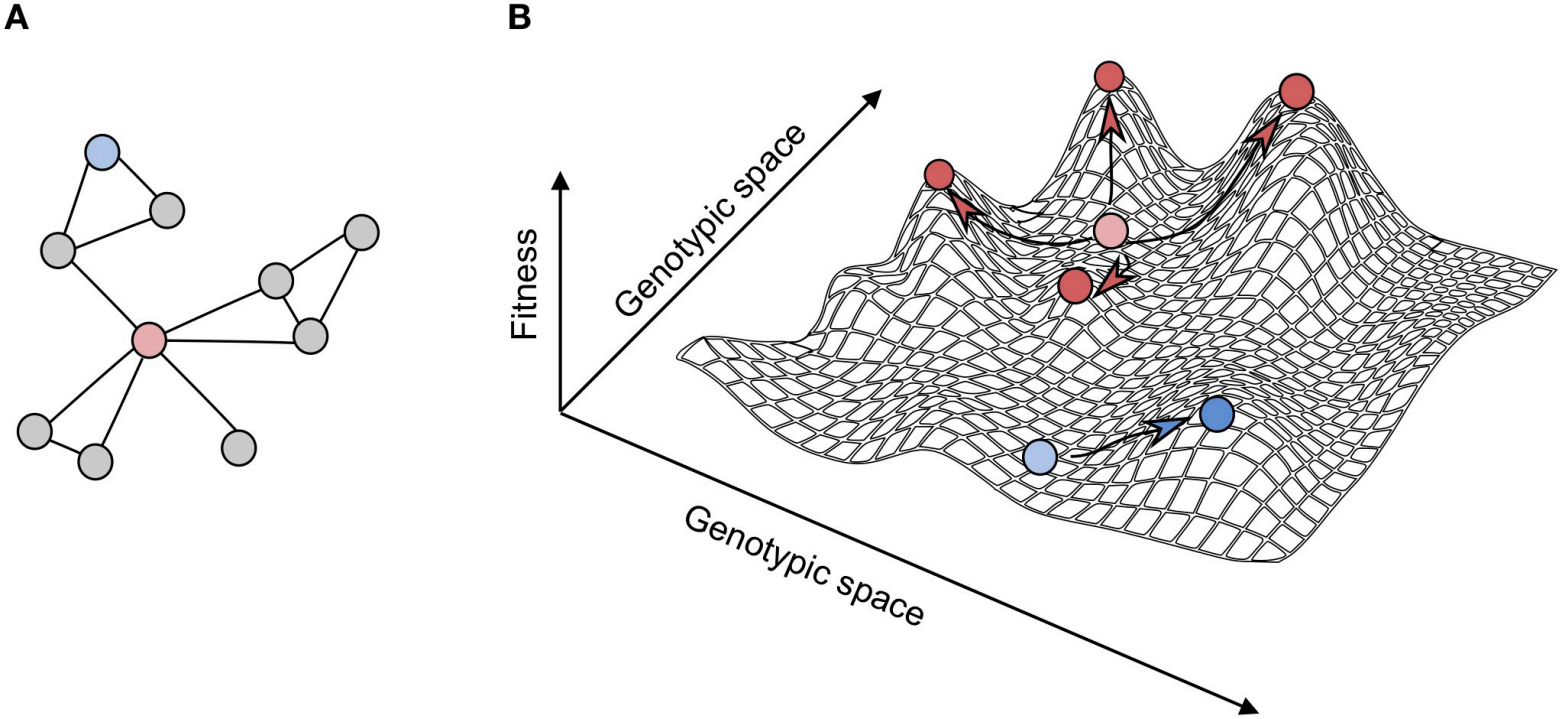
Network architecture determines evolutionary trajectories. (A) Simplified schematic of a gene network. The red node is a hub gene, and the blue node is a gene at the edge of the network. (B) Fitness landscape and possible evolutionary trajectories. Perturbing a hub gene (red) or a peripheral gene (blue) can both lead to a decrease in fitness, but the number of available evolutionary trajectories (arrows) is higher when a hub gene is perturbed.

Altogether, these new findings indicate that perturbations in hubs can speed up evolution, rather than being obstacles for evolution. In contrast to most studies that look at the link between connectivity and the rate of evolution, Koubkova-Yu and colleagues use experimental evolution to explicitly explore the importance of network architecture in evolution. First, they show that although a mutated hub generally results in fitness defects, it can improve growth in certain environments (e.g., salt stress). This means that in stress conditions, in which a hub does not function properly, a mutation can have a large beneficial effect on fitness even though it might have a detrimental effect on its ability to form protein–protein interactions. Second, the authors show that adaptation to mutations in a hub can be fast but tends to happen in the periphery of the network and not in the hub itself. Taken together, this might indicate that changes in hub proteins could offer a way to quickly change the phenotypic profile and help an organism to adapt to a particular environment.

However, it is important to also note a few pitfalls and unanswered questions. Firstly, replacing a complete hub protein with an ortholog is unlikely to be a common scenario in natural evolution. A spontaneous mutation in a hub may have very different and more negative fitness effects, which could imply an evolutionary dead end. Second, *HSP90* is in many regards a special hub because it acts as a molecular chaperone. Other studies have shown that solely changing the levels of Hsp90 alters the relation between genotype and phenotype, possibly affecting evolutionary processes [11,22]. In addition, because only a single hub gene was studied by Koubkova-Yu and colleagues, it is difficult to draw general conclusions about the role or importance of network architecture in evolution. Some previous studies focused on linking the rate of evolution (adaptability) to the initial fitness of the genotype rather than investigating the role of network architecture on evolvability. These studies show that lower initial fitness leads to faster fitness improvements through evolution (rule of declining adaptability) [23]. Therefore, an interesting and important yet difficult challenge is to disentangle to what extent fitness, the fitness landscape, or the underlying molecular network shape evolution.
